# The impact of climate change on airborne and droplet-borne diseases and hospital response measures

**DOI:** 10.3389/fpubh.2026.1809465

**Published:** 2026-09-04

**Authors:** Po-Hsiu Huang, Jheng-Yi Yeh, Jia-Jen Jang, Hsien-Po Huang, Ting-Kuang Yeh, Wei-Hsuan Huang, Chien-Hao Tseng, Yan-Chiao Mao, Yi-Fang Ho, Yu-Fen Chen, Yu-Yueh Shih, Pei-Chun Pan, Chun-Hsi Tai, Yu-Hsia Hen, Hsin-Yi Hung, Pei-Hsuan Huang, Po-Yu Liu, Chia-Wei Liu

**Affiliations:** 1 Division of Infectious Diseases, Department of Internal Medicine, Taichung Veterans General Hospital, Taichung, Taiwan; 2 Infection Control Center, Taichung Veterans General Hospital, Taichung, Taiwan; 3 Genomic Center for Infectious Diseases, Taichung Veterans General Hospital, Taichung, Taiwan; 4 PhD Program in Molecular Biology, Institute of Molecular Biology, National Chung Hsing University, Taichung, Taiwan; 5 School of Medicine, National Yang Ming Chiao Tung University, Taipei City, Taiwan; 6 Department of Medical Toxicology, Taichung Veterans General Hospital, Taichung, Taiwan; 7 School of Medicine, National Defense Medical University, Taipei City, Taiwan; 8 Department of Post-Baccalaureate Medicine, College of Medicine, National Chung Hsing University, Taichung, Taiwan; 9 Doctoral Program in Translational Medicine, Rong Hsing Translational Medicine Research Center, National Chung Hsing University, Taichung, Taiwan; 10 Graduate Institute of Biomedical Engineering, National Chung Hsing University, Taichung, Taiwan

**Keywords:** climate resilience, health systems, hospital preparedness, infection control, respiratory viruses

## Abstract

**Objectives:**

This review synthesizes quantitative evidence on how climate change modulates airborne and droplet-borne disease transmission and assesses hospital resilience strategies.

**Methods:**

A narrative review of global data (2000–2025) was conducted, focusing on pathogen kinetics and post-disaster hospitalization burdens.

**Results:**

Climate change alters disease dynamics by extending pathogen viability and creating prolonged transmission windows. Flood exposure increases respiratory hospitalization risk by 30% and infectious disease hospitalization risk by 26%, with burdens persisting for 60–90 days. Heat exposure increases COPD risk by 1.47% for every 1 °C above thermal thresholds. Mechanistically, low humidity favors persistent airborne droplet nuclei, whereas high humidity enhances transmission via larger droplets that require close contact. Current hospital infrastructure remains vulnerable to these compound climate-pollution crises.

**Conclusion:**

Conventional acute-phase disaster models are insufficient. Resilience requires a paradigm shift toward “climate-integrated” infection control. Critical strategies include integrating meteorological data into predictive surveillance systems and using ventilation, upper-room UVGI, and portable air cleaning as complementary engineering controls.

## Introduction

Climate change constitutes a profound and escalating threat to global health, extending far beyond the direct impacts of heatwaves and extreme weather events (1). Growing evidence illuminates the multifaceted ways climate change influences the epidemiology and transmission of infectious diseases. Among these, airborne and droplet-borne diseases represent a significant and persistent public health burden globally. Characterized by transmission through respiratory particles, these diseases are particularly sensitive to the environmental alterations driven by climate change (2). However, these effects are not uniformly negative; depending on the pathogen, geographical setting, and season, climate change may intensify, attenuate, or redistribute transmission patterns.

Variations in fundamental climatic parameters, notably temperature and humidity, directly affect pathogen survival and infectivity outside the host, thereby influencing the likelihood of transmission (3). Furthermore, the increasing frequency and intensity of extreme weather events—such as floods, heatwaves, and droughts—indirectly impact disease dynamics by disrupting ecosystems, modifying human behavior (e.g., displacement leading to crowding), and compromising critical public health infrastructure (4).

Recent reviews have examined viral respiratory infections, influenza-specific epidemiology, and broader climate–infectious disease relationships (2, 5, 6). However, these studies have generally provided less integration between pathogen-level transmission mechanisms and the operational preparedness of hospitals. This review goes beyond qualitative description to synthesize recent quantitative evidence on these impacts and current scientific understanding of climate change’s effects on airborne and droplet-borne disease transmission, distribution, seasonality, and severity. Accordingly, the central question of this review is: how does climate change modify airborne and droplet-borne disease dynamics, and which hospital response measures should be prioritized? We elaborate on how climate variability reshapes the epidemiology of respiratory infections and assess hospital preparedness. Drawing on global evidence, including Asia’s experience, we propose evidence-based measures to bolster healthcare resilience against climate-driven infectious disease threats and identify key challenges to address for effective adaptation and response.

## Methods

This study was conducted as a narrative review synthesizing evidence on the relationship between climate change, airborne and droplet-borne diseases, and hospital preparedness. This work does not constitute a systematic review and was not registered in PROSPERO; no pre-specified protocol, dual independent screening, or formal risk-of-bias assessment was performed.

The literature synthesis proceeded in two complementary stages. First, PubMed (MEDLINE) was searched on 16 May 2026 using a Boolean string combining three thematic blocks—climate and environmental exposures, respiratory infectious pathogens, and hospital or health-system contexts—restricted to English-language publications between 1 January 2000 and 31 May 2025. The complete search string, including MeSH terms, field codes, and filters, is provided in Supplementary material S1. This search yielded 936 records and captured primary epidemiological and mechanistic evidence linking environmental factors to respiratory infection outcomes. Second, to develop a comprehensive framework for hospital preparedness and health-system adaptation, we conducted targeted hand-searching of reference lists from systematic reviews, scoping reviews, and clinical guidelines identified in the primary search, supplemented by review of authoritative documents from the World Health Organization (WHO), Intergovernmental Panel on Climate Change (IPCC), U.S. Centers for Disease Control and Prevention (CDC), and regional public health agencies (e.g., Taiwan’s Communicable Disease Control Medical Network). Approximately 20 additional records were identified through this supplementary approach. To incorporate important developments published during manuscript revision, a targeted citation update of key references was additionally conducted through 16 May 2026.

**Figure 1:**
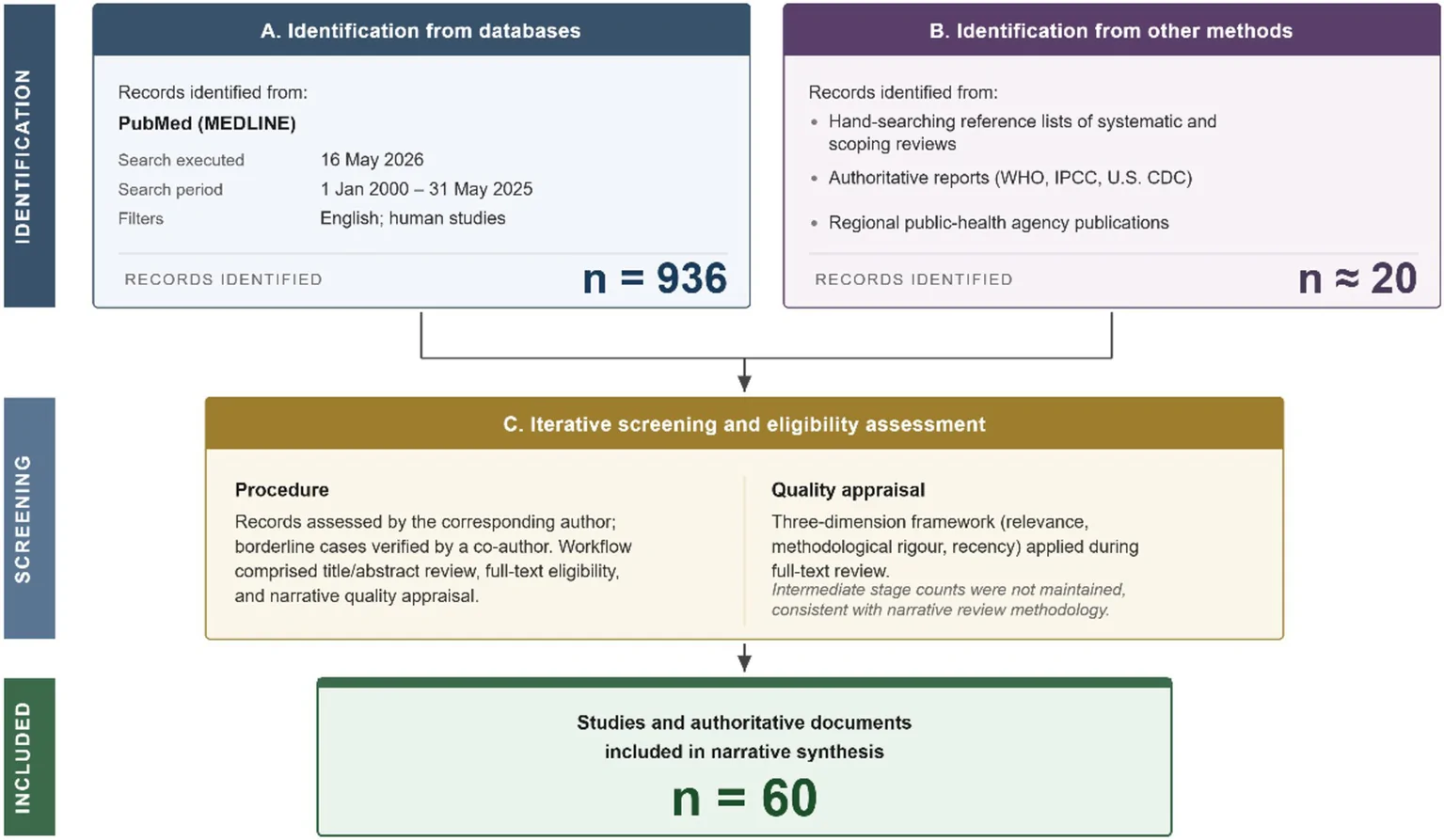
PRISMA-style flow diagram of the literature search and selection process for this narrative review.

Search terms integrated concepts pertaining to climate change (“climate change,” “global warming,” “extreme weather,” “air pollution”), respiratory infections (“airborne,” “droplet-borne,” “influenza,” “tuberculosis,” “measles,” “COVID-19,” “RSV”), and health system responses (“hospital preparedness,” “infection control,” “infrastructure resilience”). The screening and inclusion workflow is illustrated in Figure 1. While this review does not claim full adherence to the PRISMA 2020 checklist, the search and screening process was documented using a PRISMA-style flow diagram to support methodological transparency.

Only peer-reviewed articles, official reports, and authoritative reviews written in English were included. Studies focusing on vector-borne or waterborne diseases were generally excluded, except where they provided essential technological frameworks (e.g., predictive modeling architectures) applicable to airborne disease surveillance. Records were assessed by the corresponding author; borderline cases regarding relevance or eligibility were verified by a second co-author. Based on these criteria, 60 studies and authoritative documents were synthesized into the review. Additional contextual, methodological, reporting, and technical sources cited during manuscript revision were not included in the core narrative synthesis count shown in Figure 1. Evidence was extracted and systematically categorized into three primary domains: (1) the mechanisms by which climate change influences disease transmission; (2) observed or projected impacts on specific respiratory diseases; and (3) hospital preparedness and adaptation strategies. This thematic framework facilitated the synthesis of global findings and region-specific experiences, particularly from Asia, to provide a comprehensive assessment of the challenges and opportunities in developing climate-resilient healthcare systems.

Although formal risk-of-bias assessment tools (e.g., ROBIS) and GRADE certainty grading are not applicable to narrative reviews, we applied a pragmatic narrative quality appraisal during synthesis. Included studies were evaluated across three dimensions: (1) study design category (e.g., randomized controlled trials, observational cohort studies, ecological or time-series analyses, and expert reviews); (2) sample size, geographic coverage, and generalizability; and (3) validity of outcome measurement. The majority of evidence linking climate variables to disease transmission derives from ecological and time-series observational studies, which support identification of associations but cannot establish individual-level causation. Readers should therefore interpret quantitative estimates reported in this review as illustrative of effect magnitudes identified in the source literature, rather than as pooled or certainty-graded estimates. The methodological implications of this approach are discussed further in the Limitations section. To support transparency in evidence classification, Supplementary Table S2 provides a structured summary of the key climate variables examined, the structural characteristics of each pathogen class, and the corresponding narrative evidence strength for each pathogen–climate pairing discussed in this review.

Records were identified through two parallel pathways: (1) a PubMed (MEDLINE) database search (*n* = 936) and (2) hand-searching of reference lists from systematic reviews and authoritative reports from the World Health Organization (WHO), Intergovernmental Panel on Climate Change (IPCC), U.S. Centers for Disease Control and Prevention (CDC), and regional public health agencies (*n* ≈ 20). Following screening and eligibility assessment, 60 studies and authoritative documents were included in the narrative synthesis. As this is a narrative review, intermediate stage counts are reported as approximate values reflecting the iterative nature of screening; the initial database hits (*n* = 936) and final included count (*n* = 60) are precise. Full search string and execution details are provided in Supplementary material S1.

## Results

### Mechanisms through which climate change impacts disease transmission

**Figure 2:**
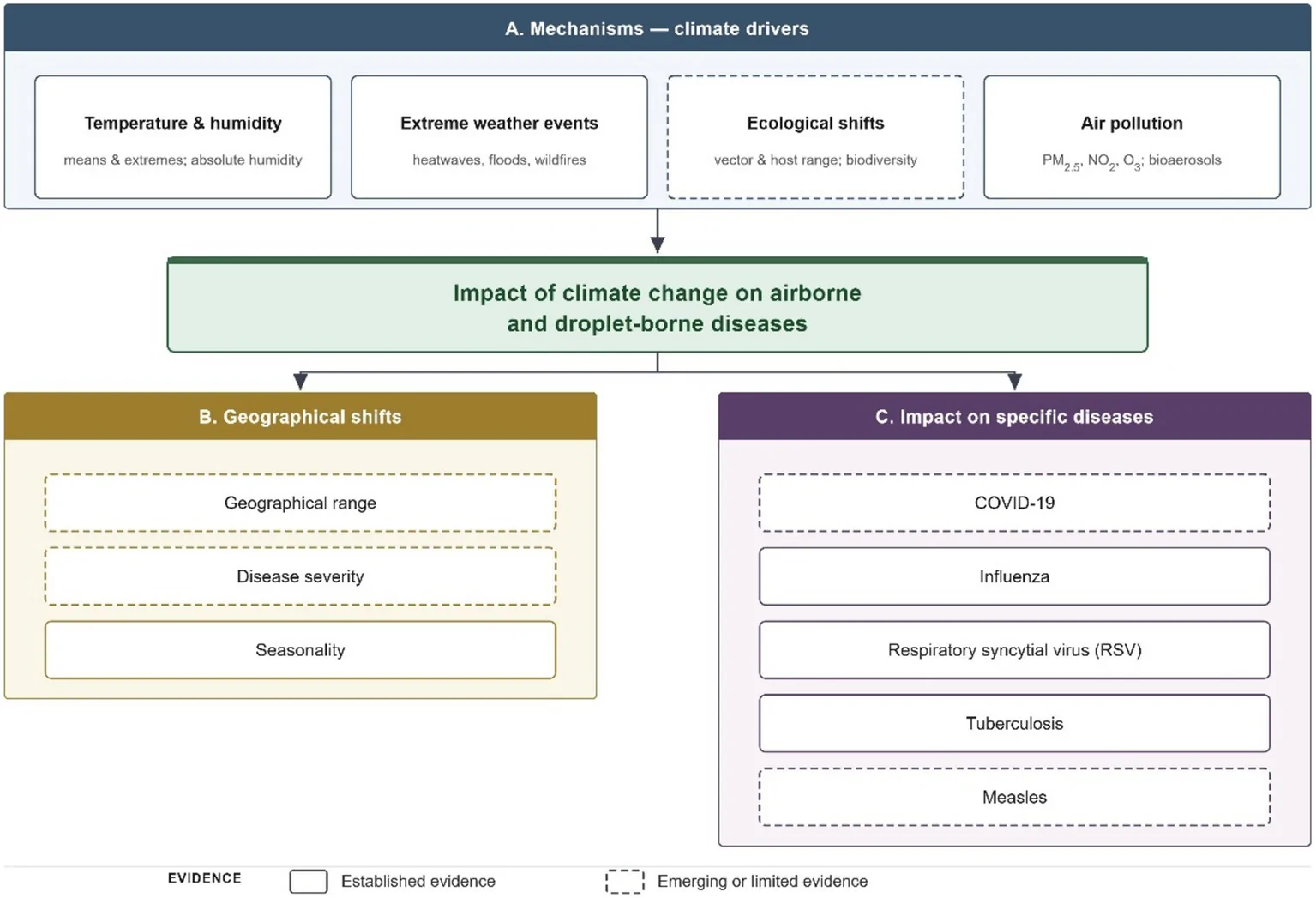
Overview of the impacts of climate change on airborne and droplet-borne diseases.

The impact of climate change on respiratory pathogens is multifaceted, driven by changes in fundamental meteorological parameters and broader environmental shifts. As illustrated in Figure 2, these mechanisms, ranging from temperature and humidity variations to extreme weather events and air pollution, collectively reshape disease transmission patterns. The following sections detail these specific pathways and their biological implications.

Overview of the impact of climate change on airborne and droplet-borne diseases. The schematic illustrates the complex interplay between climate drivers—including temperature, humidity, extreme weather events, ecological shifts, and air pollution—and disease dynamics. It details how these factors influence geographical distribution, seasonality, and severity, specifically affecting pathogens such as coronavirus disease 2019, influenza, respiratory syncytial virus, tuberculosis, and measles. Solid arrows indicate associations supported by established quantitative evidence; dashed arrows indicate emerging or limited evidence requiring further investigation.

#### Temperature and humidity: regulating pathogen survival and transmission

Temperature and humidity are pivotal environmental factors that directly influence the viability of airborne and droplet-borne pathogens outside human hosts (3). These parameters dictate the longevity of viruses, bacteria, and fungi in aerosols and on surfaces, and modulate the physical properties of respiratory droplets that mediate spread. For many viruses, including influenza (7) and measles (8), survival in aerosolized form often exhibits an inverse relationship with relative humidity. Recent studies quantify this relationship, showing that for Influenza A virus, the 99% inactivation time (T99) exceeds 5 h at RH < 30%, but drops dramatically to approximately 15–20 min at intermediate humidity (40–60%) (9). It should be noted that these values were obtained under controlled aerosol chamber conditions; real-world ward environments involve confounding variables—including aerosol composition, organic load, and air turbulence—that may alter inactivation kinetics.

However, the relationship is not always linear; some viruses display a U-shaped survival curve with respect to humidity, thriving at very low and very high humidity levels but less so at intermediate levels (3). Furthermore, absolute humidity, reflecting the actual water vapor content in the air, may be a more robust predictor than relative humidity for the survival and transmission of some viruses, such as influenza (10), particularly in temperate zones, as it directly relates to droplet hydration and pathogen viability.

Conversely, certain bacterial pathogens, specifically *Mycobacterium tuberculosis*, display complex interactions with environmental conditions. Experimental studies have demonstrated that *M. tuberculosis* can remain viable following aerosolization, although the direct effects of humidity on its aerosol survival remain incompletely characterized (11). Transmission is also strongly influenced by droplet evaporation kinetics. In low relative humidity environments (e.g., <40%), respiratory secretions undergo rapid desiccation (12), transforming into smaller “droplet nuclei” (<5 μm) within fractions of a second. During this desiccation process, NaCl and other solute concentrations may increase substantially above physiological levels as water evaporates, imposing osmotic stress on enclosed pathogens. Concurrently, mucin glycoproteins—constituting approximately 2–5% of respiratory mucus by weight—become concentrated and undergo a sol-to-gel phase transition that can temporarily buffer pathogen exposure to osmotic extremes. Upon complete desiccation, pathogen viability within the resulting droplet nucleus is governed principally by water activity (13). These nuclei, which are critical for TB transmission, possess negligible settling velocity and remain suspended in air currents for extended periods, significantly expanding the spatial radius of infection risk.

**Figure 3:**
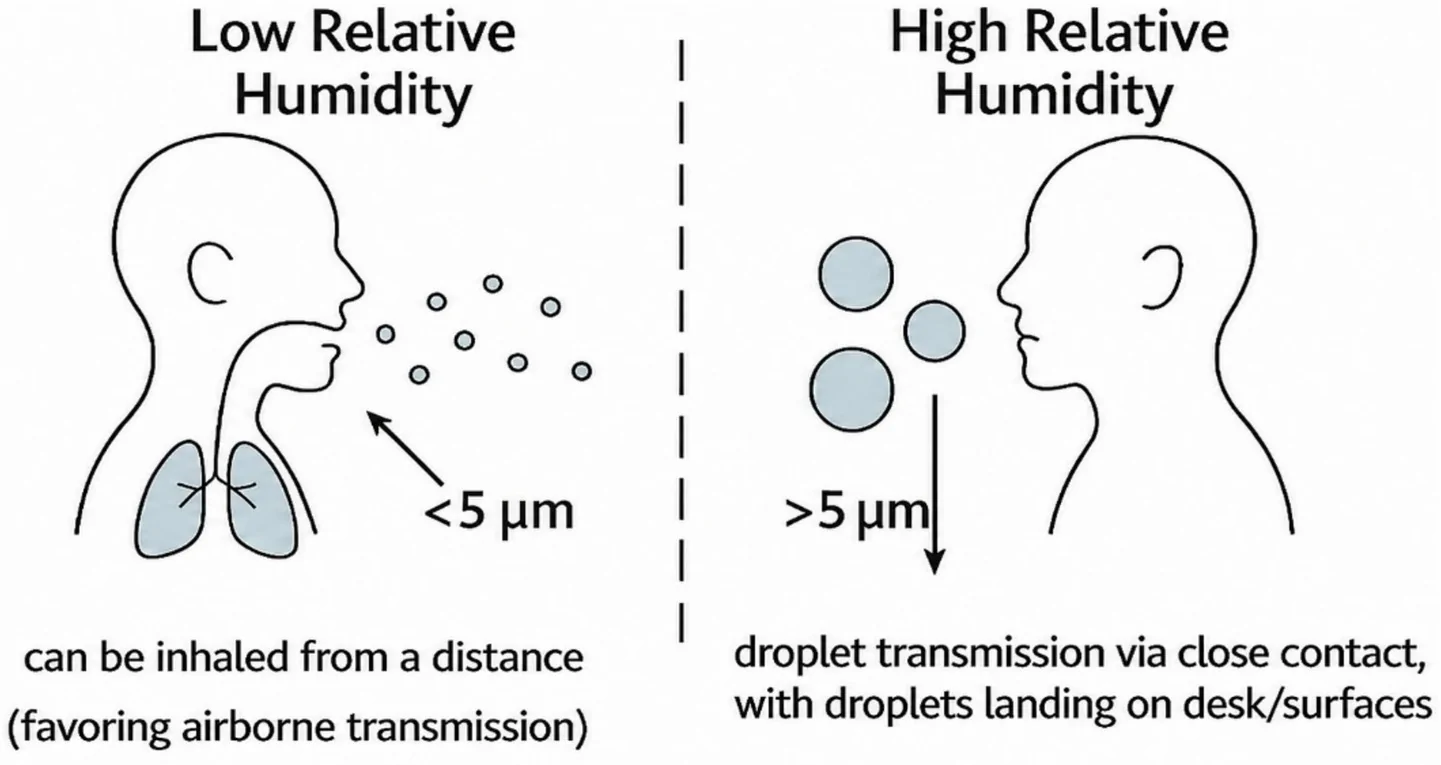
Impact of relative humidity on droplet size and respiratory transmission pathways.

In contrast, high relative humidity retards evaporation, maintaining droplets in a larger, heavier state (>5 μm) that follows a ballistic trajectory and settles quickly under gravity. While this limits the range of airborne spread, it favors droplet transmission via close contact. This mechanism, illustrating the critical role of humidity in modulating transmission potential, is depicted in Figure 3. This biophysical bifurcation underscores the importance of indoor humidity control (optimally targeting 40–60%) as an active engineering measure in hospital wards to mitigate transmission risks.

The magnitude and direction of these humidity-mediated effects differ fundamentally according to pathogen structural class, a distinction with direct implications for targeted infection control. Enveloped RNA viruses, including influenza, SARS-CoV-2, and RSV, possess a lipid bilayer membrane that is susceptible to humidity-mediated disruption; osmotic stress at low RH destabilizes the envelope, while the protective protein-salt matrix formed during rapid droplet desiccation can paradoxically slow further inactivation at very low RH extremes (<20%). *Mycobacterium tuberculosis*, by contrast, is shielded by a dense waxy mycolic acid outer layer that confers substantial environmental stability independent of humidity; its climate sensitivity operates primarily through the droplet evaporation kinetics described above, rather than through direct envelope degradation. Fungal conidia, such as *Coccidioides* species and *Candida auris*, possess rigid polysaccharide cell walls with no lipid envelope, conferring broad humidity tolerance; for these pathogens, ambient temperature rather than humidity is the dominant selective pressure driving environmental persistence and emergence. Recognition of these structural distinctions is essential for designing pathogen-specific environmental control protocols within hospital settings.

Biophysical bifurcation of transmission pathways mediated by relative humidity. Low humidity (<40%) drives droplet nuclei formation favored by airborne pathogens like TB, while intermediate humidity (40–60%) minimizes viral stability for pathogens like Influenza.

Beyond pathogen survival, extreme temperatures also impair host defenses. The thermal sensitivity of the respiratory system is quantifiable. A multicounty Medicare study found that every 10 °F increase in daily mean summer temperature was associated with a 4.3% increase in same-day emergency respiratory hospitalizations (14). Specific pathologies exhibit distinct thresholds; for instance, Chronic Obstructive Pulmonary Disease (COPD) admissions increase by 1.47% for every 1 °C above local thermal thresholds (e.g., 23.2 °C), driven by the synergistic effects of heat stress and heat-induced air quality degradation (e.g., ground-level ozone) (15).

#### Extreme weather events: direct and indirect impacts on transmission

The escalating frequency and intensity of extreme weather events disrupt environmental norms and human behavior, significantly altering patterns of infectious disease transmission, including airborne and droplet-borne illnesses (4). Events like floods (16), heatwaves (17), and droughts (18) exert both direct and indirect effects, posing substantial public health challenges. The principal mechanisms and quantitative respiratory-health impacts associated with floods, heatwaves, and droughts are summarized in Table 1.

**Table 1:** Impacts of extreme weather events on respiratory disease transmission.

Event type	Mechanism of impact	Observed quantitative risk (recent evidence)
Floods (16)	Crowded shelters, mold proliferation, and disrupted sanitation	+30% risk of respiratory hospitalization (RR 1.30); +26% risk of infectious disease hospitalization (RR 1.26); Elevated risks persist for 60–90 days post-exposure (19).
Heatwaves (17)	Airway inflammation, ozone interaction, immune impairment	+4.3% respiratory admissions per 10 °F rise in daily mean temperature (14); +1.47% COPD risk per 1 °C increase above threshold (15).
Droughts (18)	Dust mobilization, concentration of pathogens in water sources	Projected expansion of climatically suitable areas for coccidioidomycosis under warming and altered precipitation patterns (20).

Quantitatively, while historical data emphasized immediate trauma, recent multi-country time-series analyses have characterized the profound and persistent burden of flooding on healthcare systems. A landmark 2025 multi-country time-series study—a design that identifies associations at the population level but cannot establish individual-level causality—analyzing over 300 million hospitalization records across eight regions, including Taiwan, revealed that flood exposure is associated with a 30% increased risk of respiratory disease hospitalizations (RR 1.30; 95% CI 1.13–1.49) and a 26% increase in infectious disease admissions (RR 1.26) (19). The consistency of these associations across geographically diverse regions strengthens their plausibility, though residual confounding by concurrent disasters or healthcare access disruption cannot be fully excluded. Crucially, this heightened risk is not transient; elevated hospitalization rates for respiratory conditions persist for up to 60 days, and for infectious diseases, up to 90 days post-disaster, challenging the traditional emergency response timeline and necessitating prolonged surge capacity protocols.

Droughts, while distinct from acute flood and heat events, exert mechanistically rich and underappreciated effects on airborne disease transmission through several converging pathways. First, temperature and precipitation influence the environmental suitability of *Coccidioides*. Climate-niche modeling projects northward expansion of endemic areas under warming scenarios (20). Second, drought conditions elevate ambient particulate matter (PM2.5 and PM10) concentrations, which impair mucociliary clearance, induce airway inflammation, and compromise alveolar macrophage function, thereby increasing host susceptibility to secondary bacterial and viral respiratory infections (21). Third, water scarcity drives population displacement and crowding around limited water sources, increasing close-contact transmission risk for droplet-borne pathogens including measles, influenza, and tuberculosis, while simultaneously disrupting hygiene practices essential for infection prevention (18). Fourth, droughts compromise agricultural productivity, contributing to undernutrition that weakens cell-mediated immunity—a particularly important driver of tuberculosis reactivation and progression (22). Collectively, these pathways indicate that drought should be regarded as a substantive, mechanistically distinct driver of respiratory disease burden, warranting equal analytical attention to that afforded floods and heatwaves in climate-health preparedness frameworks.

#### Ecological shifts: altering environmental conditions and interactions

While primarily impacting vector-borne diseases, climate-driven ecological transformations can indirectly influence airborne and droplet-borne illnesses (23). Changes in land use, biodiversity loss, and altered migration patterns of reservoir hosts can influence zoonotic spillover events, including those involving respiratory pathogens. Furthermore, climate-induced displacement or changes in agricultural practices can alter human density and proximity to potential reservoirs, indirectly affecting transmission risks (24).

#### Air pollution: a synergistic threat

Exposure to particulate matter and ozone can damage the respiratory epithelium, impair mucociliary clearance and macrophage function, and promote airway inflammation, thereby increasing susceptibility to respiratory infection (21, 25). Emerging evidence suggests that air-pollution particles may also act as carriers for airborne pathogens, although this mechanism requires further investigation (25).

#### Evolutionary pressures: does climate change influence pathogen adaptation?

A critical area of scientific debate is whether climate warming acts as a selective pressure for increased pathogen virulence. The “thermal adaptation hypothesis” finds robust support in mycology, exemplified by the simultaneous global emergence of Candida auris. The thermal-adaptation hypothesis proposes that environmental warming may have selected for greater thermotolerance in independently emerging *C. auris* clades, potentially facilitating breach of the mammalian thermal barrier (26). For respiratory viruses, however, direct evidence that climate change selects for increased intrinsic virulence, accelerates mutation rates, or promotes antigenic escape remains limited (2, 6). Climate-related changes may alter transmission opportunities through shifts in seasonality, crowding, and population displacement, but these epidemiological effects should not be interpreted as evidence of direct viral evolutionary selection. Accordingly, enhanced genomic and epidemiological surveillance is warranted, while a direct causal link between climate change and respiratory-virus hyper-virulence remains unproven.

### Shifts in geographical distribution, seasonality, and severity

#### Shifts in geographical range

Climate change is altering the suitable habitats for pathogens and their hosts, leading to shifts in the geographical distribution of airborne and droplet-transmitted diseases. Some endemic regions may become less favorable, while new areas become permissive.

For instance, the fungal pathogen *Coccidioides immitis/posadasii*, which causes coccidioidomycosis (Valley fever) and is traditionally found in the arid southwestern U.S., Mexico, and parts of Central and South America, has shown evidence of northward expansion, potentially linked to changes in temperature and precipitation patterns that affect soil conditions (27). While primarily acquired through spore inhalation from the environment rather than person-to-person transmission, its spread exposes naive populations to risk.

#### Alterations in seasonality

Climate change disrupts the predictable seasonal patterns of many infectious diseases, complicating public health surveillance, resource allocation, and prevention strategies (e.g., vaccination campaigns).

Respiratory Syncytial Virus (RSV), typically peaking in winter in temperate regions, has shown altered timing, with some studies suggesting earlier seasonal declines linked to rising temperatures (2). Similarly, influenza seasonality (timing, duration, intensity) is expected to change under altered temperature and precipitation regimes, potentially leading to less predictable outbreaks (2).

Importantly, these effects are not uniformly adverse. Warmer conditions may attenuate influenza peak intensity (28); precipitation and humidity may reshape RSV transmission in tropical settings (29); and warming may shorten the RSV season in some temperate settings (30). However, these potential benefits may be offset by altered outbreak timing, geographical redistribution, and reduced predictability. Climate change should therefore be understood as reshaping, rather than uniformly increasing, respiratory infectious-disease risk.

#### Potential increase in disease severity

While complex and multifactorial, concerns exist that climate change could indirectly influence disease severity. Environmental stressors, particularly heat and air pollution, may worsen underlying chronic respiratory disease and increase the clinical vulnerability of affected populations (31). Furthermore, although altered environmental conditions may affect transmission opportunities, direct evidence that climate change increases the intrinsic virulence of influenza or other respiratory viruses remains limited (2, 6).

As detailed in the Evolutionary Pressures section, the mechanistic debate centers on whether climate change acts primarily through pathogen-side selection or host-side immune impairment. Distinguishing these pathways carries direct policy implications: a pathogen-side dominant mechanism prioritizes enhanced genomic surveillance and rapid vaccine reformulation, whereas a host-side dominant mechanism elevates interventions addressing heat stress, air pollution exposure, and nutritional vulnerability. Current evidence more consistently supports the host-side pathway—mediated by heat-induced suppression of mucosal immunity, ozone-driven impairment of alveolar macrophage function, and malnutrition secondary to climate-affected food systems—though both pathways may operate simultaneously under compound climate-health crises.

### Impact on specific diseases

#### Coronavirus disease 2019

The COVID-19 pandemic highlighted the complex interplay between environmental factors and respiratory virus transmission (32), with some analyses linking extreme weather events and periods of low sunlight to higher COVID-19 mortality (25, 33)—though isolating climate signals from concurrent behavioral and immunological confounders proved methodologically challenging, as detailed below.

The role of climate in shaping early SARS-CoV-2 transmission dynamics remains a subject of ongoing scientific debate. Several studies reported negative associations between SARS-CoV-2 transmission and temperature or humidity, consistent with patterns observed for other enveloped respiratory viruses (32). However, these analyses were substantially confounded by the simultaneous deployment of non-pharmaceutical interventions, heterogeneous testing and surveillance capacity across regions, varying population density, and the near-universal absence of pre-existing immunity in 2020 (25). Although climate may exert a modest modulatory effect, the current evidence base does not permit definitive conclusions regarding its relative contribution to early SARS-CoV-2 transmission dynamics (25, 32). Potential variant-specific differences also remain uncertain. The pandemic crucially underscored the importance of indoor ventilation, a factor indirectly influenced by climate change through increased reliance on air conditioning, which can reduce fresh air exchange if not managed properly (21).

#### Influenza

Influenza transmission is generally most efficient under cool, dry conditions, explaining its typical winter seasonality in temperate climates (7, 34). Low temperatures and low humidity favor viral stability in aerosols and on surfaces, facilitating transmission both indoors and outdoors.

Climate change is projected to alter influenza dynamics (28). Warmer winters might affect outbreak timing and intensity, while changes in precipitation could influence indoor humidity levels and human behavior (e.g., crowding indoors). Maintaining indoor relative humidity between 40 and 60% has been suggested as a potential non-pharmaceutical intervention to reduce influenza virus viability and transmission in indoor settings (35).

#### Respiratory syncytial virus

Respiratory Syncytial Virus (RSV), a major cause of pediatric lower respiratory tract infections (36), exhibits climate-linked seasonality. Temperate regions typically experience winter peaks (30, 36), whereas RSV transmission in tropical settings may be associated with precipitation, temperature, and humidity (29, 36).

Climate change may shorten the RSV season in some temperate areas (30), whereas changes in precipitation and humidity may reshape transmission in tropical settings (29). Low absolute humidity is a key driver in temperate zones, whereas factors related to rainfall appear more critical in the tropics (36).

#### Mycobacterium tuberculosis

While *Mycobacterium tuberculosis* (TB) transmission is primarily driven by socioeconomic factors and healthcare access, climate change acts as a threat multiplier (22). It can exacerbate underlying drivers of the TB epidemic, including undernutrition (due to impacts on agriculture), poverty, and population displacement caused by extreme weather events (22). Climate change also contributes to air pollution, which impairs lung health and immune function, increasing susceptibility to TB infection and progression to active disease (22).

Direct links between specific climatic factors and TB transmission are complex; nonlinear and delayed associations with temperature and relative humidity have been reported, although their direction and magnitude vary across settings (37).

#### Measles

Measles transmission exhibits seasonality, often peaking in late winter/early spring in temperate zones, though patterns are more variable in the tropics. Environmental associations vary by setting. A Bangladesh study found measles-like disease incidence to be positively correlated with maximum temperature and negatively correlated with average minimum temperature and annual rainfall (38), whereas a Guangzhou time-series study identified nonlinear associations with temperature and relative humidity, with low relative humidity associated with increased measles morbidity (39). Climate-related disruptions (e.g., displacement, damaged health infrastructure) can also severely impact vaccination campaigns, increasing population vulnerability.

### Current hospital response measures

#### Unique challenges in hospitals

Hospitals are critical infrastructure but also represent high-risk environments for airborne and droplet-borne disease transmission, especially under climate change pressures (21). The concentration of vulnerable patients (older adults, immunocompromised individuals, those with comorbidities) alongside healthcare workers creates fertile ground for nosocomial outbreaks. Climate change can exacerbate this by increasing the baseline frequency or intensity of community outbreaks, leading to surges that strain hospital capacity, and by directly impacting hospital operations through extreme weather. It is important to note, however, that outdoor climate extremes do not translate directly to indoor hospital environments. Modern building management systems (BMS), mechanical HVAC infrastructure, thermal insulation, and architectural design features can substantially attenuate the outdoor climate signal, maintaining relatively stable indoor temperature and humidity even during extreme weather events in well-resourced facilities. In contrast, hospitals relying on natural ventilation—which are more common in low- and middle-income settings—exhibit a much tighter correlation between outdoor climate conditions and indoor microclimates. This heterogeneity underscores the importance of direct in-ward environmental monitoring, rather than inference from outdoor meteorological station data, when designing and evaluating climate-adaptive infection control protocols.

#### Existing hospital emergency preparedness plans

Most hospitals have existing emergency preparedness plans outlining protocols for various crises, including infectious disease outbreaks. These typically cover infection prevention and control (IPC), surge capacity management (staff, beds, equipment), resource logistics (supply chain), and communication strategies. However, a critical gap is often the extent of the explicit integration of climate change considerations. Traditional plans may not adequately account for altered disease seasonality (5), novel geographic distributions, the potential for compound crises (e.g., heatwave simultaneous with a respiratory outbreak), or the direct physical impacts of extreme weather on hospital infrastructure and accessibility (40).

#### Additional challenges posed by climate change

Climate change introduces complexities beyond outbreak scenarios. Concurrent health crises are a major concern – imagine managing a flood evacuation or a surge of heatstroke patients simultaneously with an influenza epidemic. Extreme weather can directly damage hospital facilities, disrupt transportation networks vital to staff, patients, and supplies (medications, food, water), and cause power outages, crippling essential services such as ventilation, diagnostics, and life support. These disruptions severely compromise the ability to mount an effective outbreak response (40). To address these compound crises, we propose a four-tier operational prioritization framework: Tier 1—life safety and continuity of power-dependent care; Tier 2—infection-control integrity, including pre-positioned PPE supplies and maintenance of isolation-room pressure; Tier 3—surge-capacity activation; and Tier 4—operational and communication continuity. This author-proposed framework is intended as a practical prioritization tool rather than a formally validated incident-command model. Pre-positioning Tier 2 resources may reduce dependence on reactive procurement during concurrent logistical disruptions.

#### Hospital response measures in Asia and Taiwan

Regions across Asia, particularly Taiwan, have accumulated extensive experience in managing large-scale infectious disease outbreaks such as SARS, H1N1 influenza, and COVID-19, often necessitating rapid adaptation and innovation (41). Taiwan’s experience, for instance, highlighted the critical value of integrated command centers, early implementation of universal masking and enhanced IPC measures, flexible bed management strategies, and community-wide communication, alongside the integration of technology for contact tracing and resource management (42, 43). These lessons, derived from managing acute outbreaks, provide a robust foundation for developing climate-resilient hospital preparedness strategies that anticipate environment-driven shifts in disease patterns. However, whereas Taiwan’s past success was anchored in strict containment, future preparedness in Asia is increasingly emphasizing predictive modeling, including efforts to translate climate-informed disease models into usable digital tools and the development of geographically granular climate-health datasets (44, 45).

The structural resilience of Taiwan’s healthcare system is anchored in the Communicable Disease Control Medical Network (CDCMN), a decentralized framework comprising six regional command centers. This structure allows for independent surge capacity activation, ensuring that a localized outbreak (e.g., in the tropical south) does not overwhelm national resources. The efficacy of this tiered system was demonstrably proven during the 2009 H1N1 pandemic, where rapid triage and antiviral distribution helped maintain a mortality rate of just 1.8 per million population—the third lowest among OECD member nations and approximately 20% of the rate observed in the United States (41). Yet resilience is not static; analysis of Taiwan’s early COVID-19 response identified regional changes in several healthcare-quality indicators, illustrating the potential indirect effects of large-scale outbreak responses on routine healthcare delivery (46). For future climate-driven threats, the CDCMN’s “modular” capacity remains critical, allowing hospitals in flood-prone regions to activate independent emergency protocols while neighboring regions provide logistical support.

### Enhancing hospital preparedness for climate-driven outbreaks

#### Strengthening surveillance and early warning systems

Effective preparedness requires robust surveillance systems that integrate climate and environmental data (temperature, humidity, air quality, extreme weather forecasts) with traditional epidemiological monitoring (e.g., syndromic surveillance) (47, 48). This integration allows for the identification of climate-sensitive disease patterns and potentially anticipates outbreaks. Hospitals should collaborate with public health agencies to access and use local climate projections and to develop predictive models tailored to their catchment areas (47, 49). Effective integration requires adherence to established data interoperability standards. The HL7 Fast Healthcare Interoperability Resources (FHIR) framework provides a RESTful architecture that can support data exchange between hospital electronic health records and external data repositories (50). Essential metadata fields for this linkage include: geographic patient encounter coordinates for spatial matching to weather station data; timestamp-linked ICD-10 coded diagnoses for syndromic surveillance; and concurrent ambient measurements of temperature, relative humidity, PM2.5, and UV index. In lower-resource settings, implementation should prioritize adaptable and user-friendly digital platforms capable of integrating locally available health and climate data, with platform selection guided by local interoperability, governance, workforce, and technical capacity (44). Implementing hospital-based early warning systems, triggered by integrated surveillance data, can provide crucial lead time to activate surge plans, reinforce IPC measures, check supply inventories, and adjust staffing before an outbreak escalates (49, 51).

#### Infection control measures

Hospitals must critically review and enhance their infection control protocols to effectively address the emerging challenges presented by climate change and its impact on infectious diseases. This involves optimizing ventilation systems to achieve sufficient air exchange rates and improve indoor air quality, thus minimizing airborne pathogen transmission risk, an essential consideration given the growing reliance on air conditioning systems, which can inadvertently reduce natural ventilation (52). Hospitals should enforce proper use of Personal Protective Equipment (PPE) and maintain sufficient stockpiles to sustain operations through outbreaks. Implementing air disinfection technologies like upper-room ultraviolet germicidal irradiation (UVGI) (53) and portable air cleaners is recommended, especially in high-risk areas such as emergency departments, isolation wards, and ICUs. Implementation of upper-room ultraviolet germicidal Irradiation (UVGI) is strongly recommended; a controlled study conducted in simulated learning environments demonstrated that upper-room UVGI can reduce airborne viral concentrations by up to 96% under specific conditions (ventilation rate: 3 ACH; UV fluence rate: 30–50 μW/cm^2^; room volume: standard classroom dimensions) (54). These results were obtained under controlled experimental conditions; performance in clinical ward environments may vary depending on room geometry, occupancy patterns, air mixing efficiency, and UV system installation parameters. Hospitals considering UVGI implementation should refer to institution-specific engineering assessments. Practical implementation of UVGI requires institution-specific engineering and safety assessment. Upper-room fixtures are generally mounted above the occupied zone, typically at heights greater than 2.1 m, and their performance depends on ceiling height, fixture output, room geometry, air mixing, installation, and maintenance (55, 56). These considerations are described in NIOSH guidance and related exposure studies (55, 56). A layered control approach may combine mechanical ventilation, upper-room UVGI, and portable HEPA filtration according to the characteristics of each clinical space. Experimental studies summarized in NIOSH guidance indicate that upper-room UVGI can provide pathogen-specific equivalent air-change rates ranging from low single digits to approximately 25 eACH under selected conditions. Performance varies substantially according to UV fluence, air mixing, room geometry, fixture configuration, and organism susceptibility (55). For portable HEPA units, the additional eACH can be estimated from the device CADR and the actual room volume (eACH = CADR/room volume, using consistent units). As an illustrative calculation, a unit with a CADR of 400 m^3^/h would provide approximately 5.3 eACH in a 75 m^3^ room under ideal mixing conditions. Actual performance may be lower because of device placement, airflow obstruction, filter loading, room geometry, and incomplete air mixing (52, 55). Maintaining indoor relative humidity within a moderate range, approximately 40–60%, has been proposed as a supplementary measure to reduce the viability or transmission of some respiratory viruses. However, the optimal target may vary according to the pathogen, climate, ventilation strategy, and building conditions (57).

#### Infrastructure resilience

**Figure 4:**
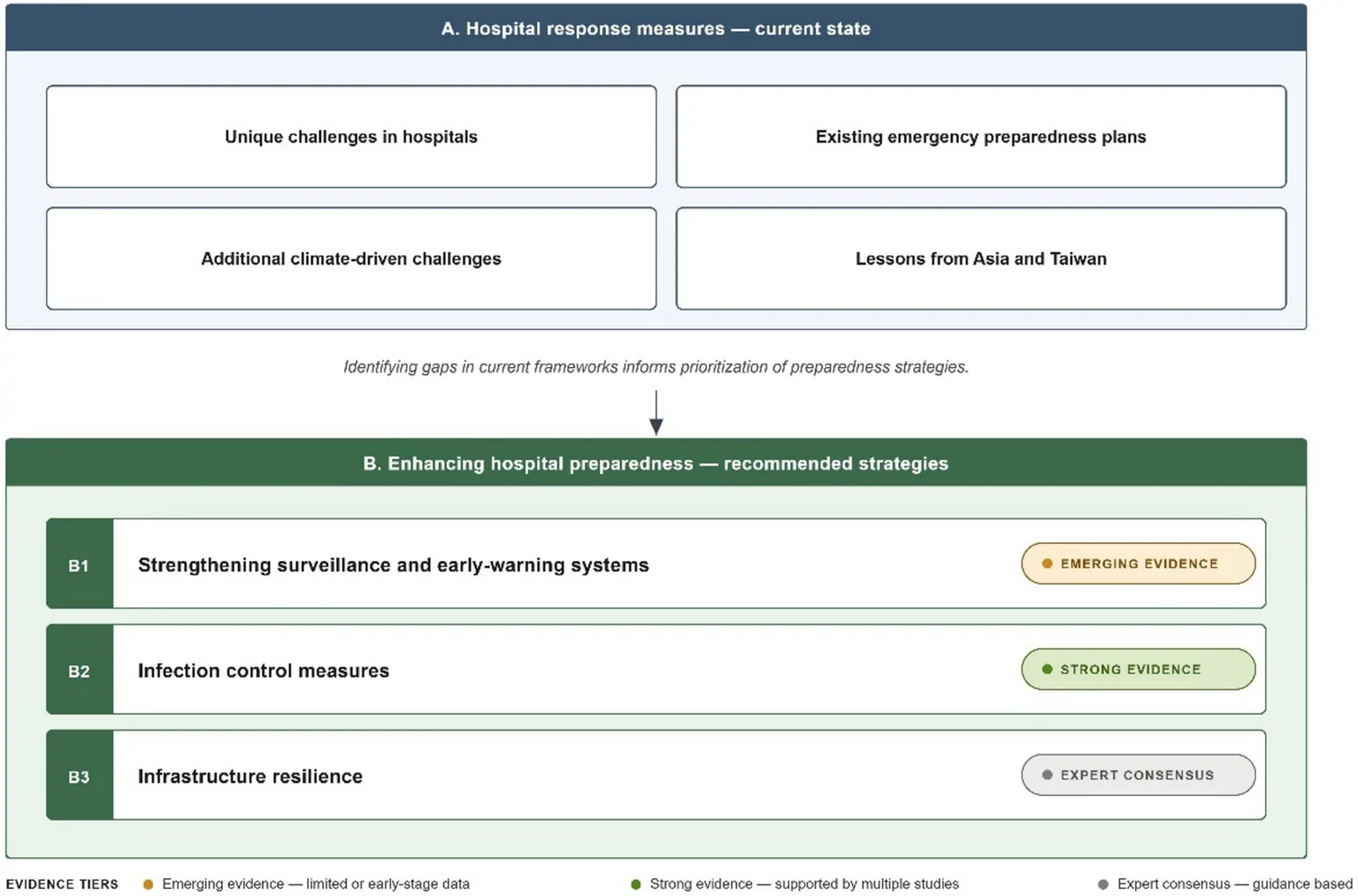
Hospital response measures and preparedness strategies for climate-driven infectious-disease threats.

Enhancing the resilience of hospital infrastructure to the impacts of extreme weather events is of paramount importance for ensuring continuity of care during climate-related emergencies. This includes investing in and maintaining reliable backup power generation systems to sustain essential hospital functions, such as lighting, ventilation, and life support equipment, during power outages caused by severe storms or prolonged heatwaves. An overview of the principal hospital vulnerabilities and corresponding preparedness strategies is presented in Figure 4.

Hospital response measures and preparedness strategies. The framework illustrates the transition from evaluating current challenges—including unique hospital vulnerabilities, existing emergency plans, and climate-specific threats—to implementing enhanced preparedness measures. It highlights the integration of regional experiences to strengthen surveillance and early warning systems, optimize infection control protocols, and bolster infrastructure resilience against climate-driven infectious disease threats. Evidence tier labels on the right side of each preparedness strategy indicate the strength of supporting evidence: “Strong evidence” denotes recommendations supported by quantitative clinical data; “Emerging evidence” denotes strategies with limited but growing empirical support; “Expert consensus” denotes recommendations based primarily on professional consensus and observational practice.

### Advanced modeling and prediction of climate-sensitive disease outbreaks

#### Current state of predictive technologies

Although an increasing number of climate-informed statistical and machine-learning models are being built to forecast climate-sensitive infectious diseases, only a small fraction have so far been converted into user-friendly digital early-warning tools that public-health practitioners can readily adopt and use (44). Datasets like EpiClim provide geographically granular epidemic and climate data, enabling models that link weather forecasts to public health risk prediction (45). Ensemble neural networks (e.g., XEWNet for dengue) demonstrate improved forecasting accuracy by incorporating climate variables, outperforming traditional methods (58). These tools offer the potential to move from reactive to proactive public health and hospital management.

#### Applications for hospital preparedness

Predictive models play a crucial role in hospital preparedness by enabling early warning systems that anticipate disease outbreaks influenced by climate factors. By forecasting potential surges in patient volume, hospitals can allocate resources effectively, ensure adequate staffing, and maintain essential supplies. Integrating climate-informed surveillance into health system planning is essential to ensure resilience and reduce health security risks (59).

#### Cross-sector collaboration and planning

Addressing the complex challenge of climate-sensitive infectious diseases requires robust collaboration across multiple sectors (59). Hospitals must engage with public health agencies, environmental protection agencies, meteorological services, urban planners, emergency management organizations, and academic researchers. Shared data platforms, joint risk assessments, coordinated response plans, and integrated policymaking are essential for developing comprehensive and effective mitigation and adaptation strategies (60).

## Discussion

### Mechanistic understanding gaps

While associations between climate variables and disease patterns are increasingly documented, significant gaps remain in fully elucidating the precise biological, environmental, and social mechanisms driving these changes, particularly for specific pathogen-climate interactions under varying socio-ecological conditions (6). Research is needed to better understand pathogen survival dynamics, host susceptibility changes, and the role of complex interactions (e.g., climate-pollution-infection) (61).

### Surveillance and modeling needs

Continued investment is needed in standardized, integrated surveillance systems that combine high-resolution climate data with real-time health information (including hospital data) (45). Improving the spatial and temporal granularity of data, enhancing data sharing protocols, and further developing and validating sophisticated predictive models (AI/ML) are critical priorities. Models need to incorporate socio-demographic factors and intervention effects alongside climate variables for greater accuracy (44, 58).

However, the operational limitations of current predictive architecture must be acknowledged. Syndromic surveillance systems based on diagnostic codes or chief complaints may have limited or setting-dependent diagnostic accuracy (51, 62). We therefore propose a two-stage confirmation approach in which an environmental trigger activates enhanced laboratory confirmation, with escalation occurring only when both environmental and laboratory signals cross predefined thresholds. Data silo barriers—organizational, technical, and legal—currently impede real-time linkage between meteorological and clinical data streams.

### Health system resilience and adaptation research

Research is vital to identify and evaluate the most effective strategies for enhancing healthcare system resilience. This includes studying the cost-effectiveness of different infrastructure adaptations [e.g., green infrastructure (63), building retrofits], evaluating workforce training programs on climate-health risks, developing best practices for surge capacity management under compound climate-health events, and assessing the effectiveness of climate-informed early warning systems in real-world hospital settings (64).

Adaptation strategies must also account for the differing financial and technological capacities of healthcare systems. High-resource facilities can prioritize BMS-integrated environmental sensors and FHIR-compatible EHR infrastructure; lower-resource settings may instead rely on community health worker sentinel surveillance networks, mobile-based syndromic reporting platforms, and solar-powered portable HEPA filtration as cost-effective alternatives. A stratified feasibility framework, rather than a single universal standard, is essential for equitable and scalable climate-health preparedness.

Implementation requirements for upper-room UVGI—including room configuration, baseline ventilation, installation, and maintenance—have been characterized in engineering and exposure studies (55, 56). Health-economic evidence specific to climate-integrated hospital infection-control protocols remains limited, and further evaluation is needed to determine its cost-effectiveness across different hospital settings.

### Policy and governance research

Effective governance frameworks are essential to support climate change adaptation within the health sector. Research should focus on identifying successful models for integrating health considerations into national and subnational climate policies (and vice versa), evaluating the effectiveness of different governance structures for intersectoral collaboration, promoting health equity in adaptation planning, and developing policies that incentivize climate-resilient healthcare infrastructure and practices (65, 66).

### Health equity considerations

The health impacts of climate-driven airborne and droplet-borne diseases are not distributed equally across populations. Socially and economically marginalized groups—including low-income communities, racial and ethnic minorities, migrant and displaced populations, older adults, individuals with pre-existing respiratory conditions, and those with limited healthcare access—bear a disproportionate burden of climate-sensitive respiratory disease (65). Several intersecting pathways drive this differential vulnerability: substandard housing with inadequate ventilation amplifies indoor transmission risk; occupational exposure among essential and outdoor workers increases contact with both pathogens and environmental stressors such as heat and air pollution; and disparities in healthcare access delay diagnosis and treatment, worsening outcomes.

Extreme weather events further widen these disparities. Flood-associated hospitalization risks varied according to socioeconomic status and population density (19). More broadly, climate-related health burdens may be greatest in communities with fewer adaptive resources (65). Post-disaster displacement concentrates vulnerable individuals in crowded shelters conducive to respiratory pathogen transmission, while disruptions to routine healthcare (e.g., vaccination programs, TB treatment continuity) compound long-term risk (16).

Within healthcare systems, equity-conscious preparedness requires more than universal protocols. Climate-resilient hospital planning should incorporate equity metrics into early warning systems (e.g., social vulnerability indices alongside meteorological data), ensure equitable distribution of personal protective equipment and infection control resources across facilities serving underserved populations, and prioritize outreach to communities historically excluded from preparedness planning. Failure to embed equity considerations into climate-health adaptation risks entrenching, rather than alleviating, existing health disparities.

The climate–respiratory disease relationships described in this review are not uniformly distributed across regions. In Sub-Saharan Africa, drought and population displacement amplify TB burden in settings with the most limited health infrastructure. In South Asia, monsoon-linked RSV peaks and urban heat island effects create compound seasonal burdens not captured in temperate models. In Latin America, the northward expansion of coccidioidomycosis represents the most clearly documented case of climate-driven pathogen geographic redistribution. In Europe and North America, heatwave-associated respiratory mortality and emerging disruption of influenza seasonality are the dominant signals. Temperature-driven geographic expansion and the heat–COPD relationship emerge as the most globally consistent findings; pathogen-specific seasonality effects remain substantially region-dependent and should be interpreted within local epidemiological and infrastructural contexts.

### Limitations

This review has several limitations. First, as a narrative review, this work does not adhere to formal systematic review methodology. Specifically, we did not perform dual independent screening, apply validated risk-of-bias instruments (e.g., ROBIS), or grade the certainty of evidence using GRADE. The review was not registered in PROSPERO. As a consequence, our synthesis is susceptible to selection bias, and the quantitative estimates presented (e.g., relative risks for flood-associated hospitalization, UVGI efficacy figures) should be interpreted as illustrative of the magnitude of effect reported in the primary literature rather than as pooled or certainty-graded estimates. Although our efforts encompassed searching multiple databases and authoritative reports to capture the breadth of available evidence, the potential for omitting relevant studies cannot be entirely ruled out. In particular, PubMed (MEDLINE) was used as the primary database without independent parallel searches in Scopus or Web of Science, and grey literature databases (e.g., Google Scholar, OpenGrey) were not searched. This single-database approach, while consistent with the narrative review design, may have led to omission of relevant studies indexed only in other sources. Second, the review was confined to English-language literature, which may underrepresent regional perspectives from non-English-speaking nations that are also substantially affected by climate change. Thirdly, the heterogeneity of study designs and outcomes within the included literature complicates direct comparisons. Numerous studies are predominantly ecological; while such designs are well-suited to detecting population-level associations between climate exposures and health outcomes, they cannot establish individual-level causation, cannot fully control for confounding at the individual level, and may reflect aggregate rather than mechanistic relationships. Finally, the rapidly advancing evidence base, particularly concerning emerging pathogens such as SARS-CoV-2, implies that certain findings may evolve as new data emerge. Despite these limitations, synthesis offers a timely and comprehensive overview of the impact of climate change on airborne and droplet-borne diseases, while emphasizing essential considerations for hospital preparedness. Future systematic reviews incorporating formal quality appraisal and evidence synthesis would be needed to establish more definitive quantitative conclusions in this field.

## Conclusion

Climate change is reshaping—and in many settings intensifying—airborne and droplet-borne infectious disease threats, challenging global public health. Quantitative evidence underscores the urgency: flood exposure alone is associated with a 30% increase in respiratory hospitalization risk and a 26% increase in infectious disease hospitalization risk, with elevated burdens persisting for up to 60–90 days post-disaster. Hospitals must urgently enhance preparedness by integrating climate data into surveillance, bolstering infrastructure resilience, optimizing infection control for new environmental contexts, and using predictive modeling. Lessons from outbreak-prone regions, like Asia, highlight the need for agility. However, robust cross-sectoral collaboration and supportive policies are crucial. Building climate-resilient healthcare systems through sustained investment, evidence-based adaptation, and explicit integration of health equity, prioritizing communities disproportionately affected by both climate change and respiratory disease burden, is imperative to protect populations amid ongoing environmental shifts.

Based on this synthesis, we propose the following priorities: (1) consider upper-room UVGI in selected high-risk zones following institution-specific engineering assessment; (2) maintain moderate indoor relative humidity, approximately 40–60%, where technically feasible and appropriate; (3) combine ventilation, UVGI, and portable HEPA filtration according to local engineering assessment; and (4) consider incorporating the author-proposed four-tier compound-crisis framework into hospital emergency planning.
