# Supplementary material for: The impact of climate change on airborne and droplet-borne diseases and hospital response measures
Source: Front Public Health. 2026 Sep 4;14:1809465. doi: 10.3389/fpubh.2026.1809465 (PMC13587622; doi:10.3389/fpubh.2026.1809465)
Supplement: Supplementary file 1 [file Supplementary_file_1.docx]

**Supplementary Material S1. Search Strategy Documentation**

## S1.1 Database and execution

• Primary database: PubMed (MEDLINE), accessed via https://pubmed.ncbi.nlm.nih.gov/

• Execution date: 16 May 2026

• Search period covered: 1 January 2000 – 31 May 2025

• Targeted citation update: Key peer-reviewed publications available through 16 May 2026 were additionally considered during manuscript revision.

• Languages: English only

**Supplementary sources:**

• Hand-searching of reference lists from systematic and scoping reviews identified through the database search

• Authoritative reports from the World Health Organization (WHO), Intergovernmental Panel on Climate Change (IPCC), and the U.S. Centers for Disease Control and Prevention (CDC)

• Regional public health agency documents (e.g., Taiwan's Communicable Disease Control Medical Network)

*Note: Scopus and Web of Science were not searched independently for this narrative review; PubMed (MEDLINE) was used as the primary database with supplementary hand-search of synthesis-level and policy literature.*

## S1.2 Full PubMed search string

(("climate change"[MeSH Terms] OR "climate change"[tiab] OR

"global warming"[tiab] OR "extreme weather"[tiab] OR

"air pollution"[MeSH Terms] OR "air pollution"[tiab])

AND

("respiratory tract infections"[MeSH Terms] OR "airborne"[tiab] OR

"droplet"[tiab] OR "influenza, human"[MeSH Terms] OR

"influenza"[tiab] OR "tuberculosis"[MeSH Terms] OR

"measles"[MeSH Terms] OR "COVID-19"[MeSH Terms] OR

"SARS-CoV-2"[MeSH Terms] OR "respiratory syncytial virus, human"[MeSH Terms] OR

"RSV"[tiab])

AND

("hospital"[tiab] OR "infection control"[MeSH Terms] OR

"health system"[tiab] OR "preparedness"[tiab] OR

"disaster planning"[MeSH Terms]))

**Filters applied:**

• Language: English

• Publication date: 2000/01/01 – 2025/05/31

• Article type: not restricted at database level; article-type judgment (e.g., excluding editorials, conference abstracts) was applied at title/abstract screening

## S1.3 Yield

PubMed records retrieved with filters applied: 936

Additional records from hand-search of reference lists and authoritative reports (WHO, IPCC, CDC, regional agencies): approximately 20

Studies and authoritative documents included in narrative synthesis: 60

*Note: Intermediate stage counts (duplicates removed, records screened, full-text assessed) were not separately maintained, consistent with the iterative nature of narrative review methodology.*

## S1.4 Inclusion criteria

• Empirical studies, modeling studies, or syntheses examining a link between a climate-related exposure (temperature, humidity, extreme weather, air pollution, seasonality) and a respiratory infection outcome

• Studies addressing hospital, health-system, or infection-control implications

• Authoritative reports from WHO, IPCC, CDC, or equivalent agencies providing essential framework or context

• Published in English between 1 January 2000 and 31 May 2025

• Key peer-reviewed publications published after 31 May 2025 were eligible only as part of the targeted citation update conducted during manuscript revision.

## S1.5 Exclusion criteria

• Studies focused exclusively on vector-borne or waterborne diseases (except where they provided essential technological frameworks)

• Studies without a respiratory or droplet-transmitted disease component

• Studies without a hospital, health-system, or preparedness implication

• Editorials or commentaries lacking substantive synthesis

• Conference abstracts or protocol-only publications

• Full text not available

## S1.6 Methodological notes

• This study was conducted as a narrative review and was not registered in PROSPERO.

• Dual independent screening was not performed; screening was conducted by the corresponding author with borderline cases reviewed by a second co-author

• Formal risk-of-bias assessment tools (e.g., ROBIS) and GRADE certainty grading were not applied

• Reporting follows PRISMA 2020 principles where applicable to narrative review design, but does not claim full adherence to the PRISMA 2020 checklist

**Supplementary Table S2.** Summary of key climate variables, pathogen structural class, narrative evidence strength, and potential hospital considerations for each pathogen–climate pairing discussed in this review.

| **Pathogen** | **Structural class** | **Key climate variables** | **Primary climate–transmission mechanism** | **Evidence strength** | **Potential hospital considerations** | **Climate-change projection** | **Key references** |
| --- | --- | --- | --- | --- | --- | --- | --- |
| Influenza A/B | Enveloped RNA virus Lipid bilayer envelope; surface HA/NA glycoproteins | RH, AH, temperature | Low RH prolongs aerosol infectivity (T99 >5 h at RH <30%); AH drives temperate seasonality; cold temperatures stabilize lipid envelope and prolong surface survival | **Strong** | Moderate indoor humidity where feasible; upper-room UVGI in selected high-risk zones; seasonal surge-capacity planning | Warmer winters may shift outbreak timing and reduce peak intensity; seasonality less predictable | [7,9,10,28,34,35] |
| SARS-CoV-2 | Enveloped RNA virus Lipid bilayer envelope; spike protein | RH, temperature, UV index, ozone | Low RH and low temperature extend aerosol half-life; UV-C inactivates rapidly; climate effects substantially confounded by NPIs and population immunity in early pandemic data | **Moderate** | Ventilation assessment; upper-room UVGI where appropriate; respiratory protection according to exposure risk; indoor humidity monitoring | Variant-specific climate responses; future interaction with novel variants uncertain | [25,32,52,57] |
| RSV | Enveloped RNA virus Lipid bilayer envelope; F and G surface proteins | AH, rainfall, temperature | Low AH drives winter peaks in temperate zones; rainfall and indoor crowding drive tropical outbreaks; fomite survival enhanced in humid conditions | **Moderate** | Pediatric ward cohorting; contact precautions; humidity control; seasonal surge capacity planning | Possible shortening of temperate outbreak season; tropical outbreak patterns may become less predictable | [29,30,36] |
| Measles virus | Enveloped RNA virus Lipid bilayer envelope; H and F surface proteins | RH, temperature (non-linear relationship) | Temperature and humidity associations vary by setting; Bangladesh data identified associations with maximum temperature, minimum temperature, and rainfall, whereas low RH was associated with increased morbidity in a Guangzhou time-series study. | **Emerging** | Vaccination campaign continuity planning; airborne precautions; negative-pressure isolation for confirmed cases | Climate-driven displacement and infrastructure disruption may expand susceptible populations | [8,38,39] |
| Mycobacterium tuberculosis | Acid-fast bacterium Waxy mycolic acid outer layer; no lipid envelope; high intrinsic environmental stability | RH, temperature, droplet evaporation kinetics | Low RH (<40%) drives rapid droplet desiccation into respirable nuclei (<5 µm); mycolic acid confers stability within droplet nuclei; climate acts as threat-multiplier via malnutrition and displacement rather than direct transmission enhancement | **Moderate** | Airborne infection isolation; ventilation assessment; upper-room UVGI where appropriate; fit-tested respiratory protection according to institutional policy | Socioeconomic disruption from climate events amplifies TB burden; direct climate–transmission links remain mechanistically complex | [11–13,22,37,55] |
| Coccidioides spp. | Fungal spores (conidia) Rigid polysaccharide cell wall; no lipid envelope; broad humidity tolerance | Soil temperature, precipitation deficit, drought severity, wind speed | Temperature and precipitation constrain environmental suitability; dry and dusty conditions may facilitate environmental conidial exposure. | **Emerging** | Respiratory protection in endemic and expanding regions; clinician awareness in naive populations; standard precautions (no isolation required) | Expansion of climatically suitable areas, including projected northward spread in the western United States. | [20,27] |
| Candida auris | Fungal yeast (nosocomial) Rigid cell wall; no lipid envelope; acquired high thermal tolerance (37°C) | Ambient temperature (thermal selection pressure) | Thermal adaptation hypothesis: proposes that environmental warming may have selected for increased thermotolerance in independently emerging clades, breaching the mammalian thermal barrier; primarily contact and nosocomial transmission | **Emerging** | Strict contact precautions; environmental disinfection with sporicidal agents; enhanced microbiological surveillance in high-risk units (ICU, haematology) | Proposed example of climate-associated fungal emergence; further adaptation under continued warming remains uncertain. | [26] |

*Key references primarily support the climate–pathogen relationships summarized in the table. Hospital considerations represent narrative recommendations and should be adapted to local infection-control guidelines, engineering assessments, and resource availability.*

*Evidence strength: Strong = supported by multiple observational and experimental studies with consistent findings; Moderate = consistent direction of effect but significant confounding present; Emerging = limited, early-stage, or predominantly ecological data only.*

**Supplementary Table S3.** Abbreviations and technical notations used in the manuscript

| Abbreviation/notation | Definition |
| --- | --- |
| ACH | Air changes per hour |
| AH | Absolute humidity |
| BMS | Building management system |
| CADR | Clean air delivery rate |
| CDC | Centers for Disease Control and Prevention |
| CDCMN | Communicable Disease Control Medical Network |
| COPD | Chronic obstructive pulmonary disease |
| COVID-19 | Coronavirus disease 2019 |
| eACH | Equivalent air changes per hour |
| EHR | Electronic health record |
| FHIR | Fast Healthcare Interoperability Resources |
| HEPA | High-efficiency particulate air |
| HVAC | Heating, ventilation, and air conditioning |
| ICU | Intensive care unit |
| IPC | Infection prevention and control |
| NPIs | Non-pharmaceutical interventions |
| PM2.5 | Particulate matter with an aerodynamic diameter ≤2.5 μm |
| PM10 | Particulate matter with an aerodynamic diameter ≤10 μm |
| PPE | Personal protective equipment |
| PRISMA | Preferred Reporting Items for Systematic Reviews and Meta-Analyses |
| RH | Relative humidity |
| RSV | Respiratory syncytial virus |
| SARS-CoV-2 | Severe acute respiratory syndrome coronavirus 2 |
| T99 | Time required for 99% pathogen inactivation |
| TB | Tuberculosis |
| UVGI | Ultraviolet germicidal irradiation |
| WHO | World Health Organization |
